# General Paresis, Practical and Clinical

**Published:** 1902-12

**Authors:** 


General Paresis, Practical and Clinical. By Robert Howland Chase, A.M., M.D., Physician-in-Chief, Friends Asylum for the Insane, Philadelphia, late Resident Physician, State Hospital, Norristown, Pa. Duodecimo, 290 pages. Illustrated. Philadelphia: P. Blakiston's Son & Co., 1902. [Price, $1.75 net.]

The appearance of a book devoted entirely to the consideration of paresis is a distinct advance in the science of psychiatry, the nearest approach being the classical monograph of Mickle, which has served as a classic since its publication.

The author is fully qualified to write on this subject having had an experience of more than twenty-five years among cases of nervous and mental diseases. Perhaps no disease of the mind is so little misunderstood among the general practitioners as paresis, and surely no disease deserves a better understanding by the "family physicians," who are the first ones to come in contact with it in their family practice. The literature on paresis is voluminous, but it is so scattered and not in a form to meet the needs of the busy practitioner. The author decided therefore to take up the subject in a methodical manner and as clearly as is possible the special features of this wonderful disease which claims its victims from every walk and station of life. The author naturally takes up the history of the development in his introductory chapter, giving the pupils of the great Esquirol the credit of bringing to light this much disputed disease.

In the succeeding chapters the synonyms and various stages are discussed, special attention being called to the prodromal stage. Short, clinical histories taken from literature are interspersed throughout the book, making it a valuable reference work as well.

Much diversity of opinion still exists as to the different varieties of paresis, the author inclining to the theory of Meynert that there are eight varieties. General paresis runs a milder and longer average course among women, remissions being less frequent. The author finds that it occurs earlier in women, and the percentage of female paretics under the age of thirty is nearly three times that of male paretics. The author asserts that conjugal general paresis has its explanation in a reciprocal syphilisation. Paresis is not an hereditary disease in the strict sense of the word but the tendency to paresis and other degenerative nerve processes is inherited and renders these unfortunates susceptible. No personal information is given regarding the author's views relating to syphilis—in fact if criticism is to be made, it is that the author rarely gives his own opinion, but quotes those of other writers. Injury to the head is recognised as a cause of paresis and several examples given of its occurrence.

The prognosis is uniformly unfavorable and according to Ziehen some years ago, there were but a dozen cases of recovery on record.

The treatment is most unsatisfactory. There is no special drug or class of drugs which can be regarded as at all remedial in character. The only hope in medication is to relieve the various conditions and to retard the progress of the disease. Iodide of potassium in small doses and for a long time is given as the most valuable drug to delay the decay of the primary neuron.

Many photographs adorn the work; full page pathological plates and diagrams are also included. On the whole, no better exposition of the subject is at hand and the author is to be congratulated on his effort. The binding, printing and paper are excellent. W. C. K.

